# Worlds apart? A scoping review addressing different stakeholder perspectives on barriers to family involvement in the care for persons with severe mental illness

**DOI:** 10.1186/s12913-017-2213-4

**Published:** 2017-05-15

**Authors:** Elleke Landeweer, Bert Molewijk, Marit Helene Hem, Reidar Pedersen

**Affiliations:** 1 Center for Medical Ethics, Institute of Health and Society, Faculty of Medicine, University of Oslo, P.O. Box 1130, Blindern, Oslo, NO 0318 Norway; 2 Department of Medical Humanities, VU University Medical Center, Amsterdam, The Netherlands

**Keywords:** Scoping review, Mental healthcare, Severe mental illness, Barriers to family involvement, Stakeholders’ perspectives

## Abstract

**Background:**

Empirical evidence shows that family involvement (FI) can play a pivotal role in the coping and recovery of persons with severe mental illness (SMI). Nevertheless, various studies demonstrate that FI in mental healthcare services is often not (sufficiently) realized. In order to develop more insights, this scoping review gives an overview of how various stakeholders conceptualize, perceive and experience barriers to FI. Central questions are: 1) What are the main barriers to FI reported by the different key stakeholders (i.e. the persons with SMI, their families and the professionals, and 2) What are the differences and similarities between the various stakeholders’ perspectives on these barriers.

**Methods:**

A systematic search into primary studies regarding FI was conducted in four databases: Medline/Pubmed, Cinahl, PsychInfo and Web of Knowledge with the use of a PICO scheme. Thematic analysis focused on stakeholder perspectives (i.e. which stakeholder group reports the barrier) and types of barriers (i.e. which types of barriers are addressed).

**Results:**

Thirty three studies were included. The main barriers reported by the stakeholder groups reveal important similarities and differences between the stakeholder groups and were related to: 1) the person with SMI, 2) the family, 3) the professionals, 4) the organization of care and 5) the culture-paradigm.

**Discussion:**

Our stakeholder approach elicits the different stakeholders’ concepts, presuppositions and experiences of barriers to FI, and gives fundamental insights on how to deal with barriers to FI. The stakeholders differing interpretations and perceptions of the barriers related to FI is closely related to the inherent complexity involved in FI in itself. In order to deal better with these barriers, openly discussing and reflecting upon each other’s normative understandings of barriers is needed.

**Conclusions:**

Differences in perceptions of barriers to FI can itself be a barrier. To deal with barriers to FI, a dialogical approach on how the different stakeholders perceive and value FI and its barriers is required. Methods such as moral case deliberation or systematic ethics reflections can be useful.

**Electronic supplementary material:**

The online version of this article (doi:10.1186/s12913-017-2213-4) contains supplementary material, which is available to authorized users.

## Background

The need to involve families in mental health care has become more recognized and underlined in the last decades [1]. Various studies have illustrated that family involvement (FI) in professional care and treatment can be beneficial for the person with severe mental illness (SMI) as well as for families (broadly defined). It can reduce the frequency of relapse and hospital admission as well as encourage compliance with medication and treatment endurance [2–5], and contribute to bettering the quality of life of family members [6, 7].

New organizational structures in mental healthcare have become more dependent on support and cooperation from the social networks of the person with SMI. Deinstitutionalization processes in mental healthcare have led to a focus on community care [8]. Family organization groups have sought attention for the position and role of families in the public debate and insist on getting more involved in professional care [9]. Furthermore, the current era of healthcare calls for a more cost-effective approach due to shrinking resources involvement of family might save public expenses or prevent the costs of mental healthcare from growing [10]. As a consequence various policy statements started to emphasize the importance of involving social networks of persons with SMI in professional care. Numerous guidelines have been developed to facilitate collaboration with family in mental healthcare [11, 12].

FI is an activity that requires collaboration and fine-tuning between three stakeholders: i.e. the professionals the person with SMI, and the family, the so-called ‘triadic collaboration.’ ‘Family’ is in this context broadly defined, i.e. any person playing a significant role in the patient’s life and social network. Despite the evidence of the beneficence of FI, it has not developed into a common practice in mental healthcare [2, 3]. Often the involvement of family in professional care is reported as poor, regarding both the uptake and the quality of FI. In practice, the implementation of FI stumbles on multiple barriers and challenges [3, 13, 14].

In this paper we carried out a scoping review of the literature that focused on both a thematic analysis of the types of barriers, as well as on who experiences which barriers: The so-called ‘stakeholder approach.’ We did not endeavor to perform a meta-analysis of quantitative outcomes. Our focus was to analyze and clarify a complex concept e.g. barriers to family involvement. [15] Initially we started this review with the idea of getting a general overview of the current barriers. During the process of collecting data, we realized that all papers included one or more stakeholder perspectives, and did not find other reviews that paid specific attention to the differences and similarities regarding the barriers reported by the various stakeholders. Stakeholders express what they consider important from their point of view in addressing barriers to FI. Their conceptions, presuppositions and experiences of barriers often reveal implicit or explicit normative thinking about barriers (e.g. conceptions of good care) [16].

Since FI is about bringing three different stakeholders together focusing on the stakeholder perspectives could add to a deeper understanding of the difficulties of conducting and implementing FI in practice (e.g. moral challenges). This again could offer a novel way to analyze, discuss and deal with the complexities that come with FI. Given the fact that involvement, cooperation and mutual understanding is at the core of FI, understanding and distinguishing differences among the various stakeholders’ perspectives on barriers to FI is a crucial starting point to understand and deal with these barriers.

We focused on FI in the context of SMI since we expected this context to include some of the most challenging and important barriers regarding FI since there is ample research on FI and its barriers during SMI and to limit our search for practical reasons. In this review, we address the following questions. First, we ask *what the main barriers are to FI regarding SMI reported by the different key stakeholders* – i.e. persons with SMI, family, and professionals. Second, we ask *what the differences and similarities are between the various stakeholders’ perspectives on these barriers.*

## Methods

### Search strategy

A systematic electronic search was conducted in the period of September to mid-November of 2015. In our strategy we followed the eight steps of Droste et al. [17]. We have chosen this systematic approach for its usefulness to explore attitudes and experiences of stakeholders (e.g. normative understanding and valuation of barriers). First the research questions were translated into a PICO scheme (population, intervention, comparison and outcome), see Table 1 below.

**Table 1:** Population, intervention, comparison and outcome (PICO)

• Population: persons with SMI, family, professionals	
• Intervention: family-involvement	
• Comparison: any or none	
• Outcome: barriers and challenges from various perspectives	

As Population adults with SMI, family and professionals were distinguished. For Intervention we formulated family-involvement (broadly defined). Comparison was not specified, and could be any or none. Outcome was described as barriers and challenges experienced from various perspectives. The second step was to build search components to develop our search strategy. We formulated three ‘search blocks’: 1) SMI, 2) FI, and 3) barriers and challenges (see additional file for a detailed description of our search strategy). As the next step, relevant search terms and synonyms were formulated and added to the blocks. The fourth step was selecting relevant information sources. Computerized databases that were searched were Medline/Pubmed, Cinahl, PsychInfo and Web of Science. The Boolean operator ‘OR’ was used within each block, while ‘AND’ was used to combine the three blocks in the search. After the search was executed and the retrieved results were collected into a reference management tool (Endnote), duplicates were removed and titles and abstracts were screened on relevance to the research question.

### Inclusion/exclusion criteria

Papers were included if they contained primary studies (any design) were published in peer-reviewed journals, abstracts were available, and were written in English. Our target group was adults experiencing SMI and receiving support from mental healthcare organizations. We excluded studies that addressed the domains of child psychiatry, forensic psychiatry or geriatric psychiatry. We did not set any year limits in our search. The studies varied in topics, content and research methods, but all included empirical results about barriers to FI in regard to care and treatment for persons with SMI. Methods of data collection in the studies varied from quantitative to qualitative approaches as well as mixed-method studies and included: in-depth interviews, quantitative assessment interviews, focus groups, document analysis, questionnaires and surveys. Only barriers presented in the result sections of the studies were included (not barriers, which were only mentioned in the introduction of discussion sections). Systematic reviews that addressed barriers regarding FI were not included because none had used our analytic approach and thus did not provide information about what types of barriers were reported by which stakeholder. However, we checked the identified systematic reviews in order to see whether we had missed relevant primary studies.

### Data extraction

**Fig. 1:**
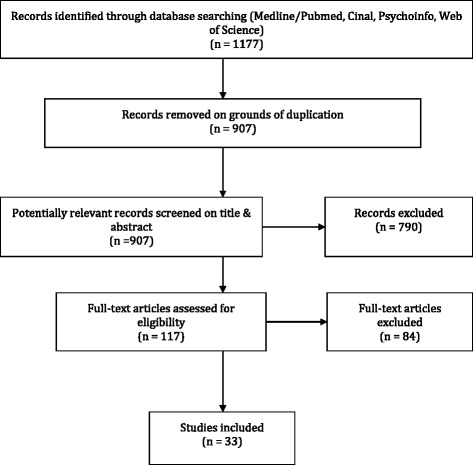
Flow diagram of the search strategy

The reference list of the papers retrieved by the electronic search in the databases contained 1177 hits. Hand searches and snowballing did not add new papers to the list. The first author (EL) screened all titles and abstracts manually after duplicates were excluded (*n* = 907). If in doubt the publication was included for further evaluation. This resulted in a selection of 258 potentially relevant studies. This second list was screened based on the titles/abstracts by the first author (EL), as well as the other authors (BM, MHH, RP). While the first author reread all the 258 titles/abstracts, the other authors divided the titles/articles between them and read the titles/abstracts of ‘their’ articles independently of the first author. In the cases where one or two of the authors either considered the article as possibly relevant to include for full-text review or was in doubt, the full-text was retrieved. This resulted in 117 full-text articles, which were then all scrutinized by the first author, while the other authors independently read and evaluated 1/3 of the full text papers each. Finally, 33 articles were included in this review Fig. 1.

### Grouping barriers

Analyzing the included articles, we sorted the barriers according to the three main stakeholder perspectives. Assuming that understanding differences in perspectives on barriers for FI among the stakeholders is important to succeed with FI, we decided to continue using this so-called ‘stakeholder approach’ to identify what was at stake for whom and detect possible domains wherein (moral) tensions could arise. The barriers of the identified studies were therefore grouped according to which stakeholder reported which barrier, in order to develop insights into which types of barriers were experienced by whom. The authors discussed how the reported barriers could be grouped and placed under which type of barrier (presented in Table 2). After that, the types of barriers reported by the different stakeholders were compared to distinguish similarities and differences between the stakeholders’ perspectives.

Some studies addressed more than one stakeholder perspective. If it was clearly specified which stakeholder addressed which barriers it was listed in the table of barriers. If two or more stakeholders expressed barriers, we listed them in more than one stakeholder group.

## Results

Thirty-three studies were included in the review [18–50]. Eight studies included barriers that were addressed by *persons with SMI* [18–24]. Eleven studies reported barriers that were addressed from the *family perspective* [21, 22, 24–32]. Twenty-five studies reported on barriers experienced by *professionals* [14, 19, 21, 22, 24–26, 33–50]. Ten studies encompassed more than one stakeholder perspective, of which seven involved all stakeholder groups [19, 21, 22, 24, 25, 28, 42], one included persons with SMI and the family perspective [23] and two involved the professional and family group [26, 37]. Twenty-three studies included only one stakeholder perspective, most often the professional perspective. Sixteen studies reported only the perspectives of professionals [33–36, 38–41, 44–50]. Five studies reported only the perspectives of family [27, 29–32] and two studies reported only on the perspectives of persons with SMI [18, 20].

Collecting the barriers addressed by the different stakeholder groups resulted in a categorization of 5 types of barriers. Three types of barriers were related to a specific stakeholder group as type that is [1–3] persons with SMI, family and professionals. Additional types were 4) barriers related to the organization of care, and 5) barriers that refer to culture and paradigms (see Table 2).

**Table 2:** Scheme of barriers –addressed by persons with SMI, families and professionals

Types of barriers →						
*Addressed by stakeholder perspectives*		**Barriers related to persons with SMI**	**Barriers related to the family**	**Barriers related to the professional**	**Barriers related to the organization of care**	**Barriers related to the culture- paradigm**
	**Persons with SMI**	Privacy concerns (18–21) Low expectations of beneficence (18) Preventing own stress (21, 22) Concerns about burdening the family (18, 19, 21) Not getting along with the family (18)	Unavailability of families (18, 23) Loss of role in families (21–23) Misunderstandings of families regarding illness/treatment (23, 24)	Professionals do not offer the opportunity (18) Disrespect (25)	Difficulty matching the families’ schedules (18)	Stigma (24, 25) Language (25)
	**Family**	Dishonesty of clients (24) Mental illness (22)	Protective attitudes (21) Discomfort regarding fellow families (26) Low expectations of effect (26, 27) Responsibilities of/in families (21, 26) Roles in families (21) Need for privacy (28)	Lack of recognition (24, 25, 28–31) Strong emphasis on confidentiality (24, 29, 31) Imposed responsibilities (31) Professionals knows best (22, 28)	Lack of resources (24, 28)	Stigma (24, 25, 32) Racial issues (24) Pre-existing worldviews (22)
	**Profes-sionals**	Confidentiality (21, 22, 35, 36, 39) Lack of consent (35, 39–42) Mental illness (37, 43, 44)	Lack of ‘suitable’ families (19, 34, 37, 41, 43, 45–47) Families not wanting to get involved (24, 40–42, 47) Difficult relationship between person with SMI and the family (37, 43, 48) Over-involvement of families (43, 49) Lack of competence in families (26, 33, 41, 49) Families’ attitudes (43) Burden for families (25, 40, 41) Power issues (22)Different cultural backgrounds of families (25, 49)	Low expectations of effect (33, 34)Motivating persons with SMI (35) Lack of competence (35–37) Lack of experience (21, 24, 26, 38) Sustainability of training (52) Inter-professional struggles (36)	Lack of time (19, 21, 22, 33, 34, 36, 40, 41, 44–46, 50) Logistical barriers (24–26, 36, 38, 40, 41, 45, 46) Priority settings (33, 34, 36, 43–45) Background expertise (34)	Medical paradigm (22, 43, 50) Management attitudes (24, 40, 46) Stigma (25, 33, 49) Past experiences (43) Language (22)

In the following, we present the types of barriers and how the different stakeholders experience and perceive them. The barriers experienced by the various stakeholders often relate to and influence each other. Stigma, for example, may influence the person with SMI to not wanting to burden their families. For each type of barrier, we describe specific aspects of the findings more in-depth to illuminate explicit differences and/or similarities in the perspectives of the various stakeholder groups.

### Barriers related to the persons with SMI

*Persons with SMI* describe having own concerns about FI. They mention privacy concerns, low expectations of positive outcomes of FI, worries about becoming stressed by FI, concerns about causing (more) family burdens, or not getting along with family [18–21]. *Families* address barriers related to persons of SMI as well. They report worries about dishonesty of persons with SMI [24], and that some phases of the mental illness could hinder FI, for example a manic episode [22]. *Professionals* address lack of patient consent for FI as barrier [35, 39–42]. The legal rules on confidentiality generally require that the professionals ask for consent from the patient before patient information may be shared with the family. Professionals also reported confidentiality as a barrier in itself [21, 22, 35, 36, 39], even in cases where the person with SMI has given permission to FI [39]. Professionals, like families, considered the mental illness of the patient as possible barrier for FI, for example in case of paranoia or aggressive behavior towards families [37, 43, 44].

The overview of ‘persons with SMI’ as type of barrier illustrates that there are differences in perspectives in whether and how *privacy* and *confidentiality* are experienced as barrier. Both persons with SMI and family address a need for own privacy. Persons with SMI express concerns that they want to keep certain issues from their families [18–21], and/or do not want to share family issues with professionals. Families might not want to share family secrets [26, 28]. Professionals report own concerns regarding the privacy and confidentiality of the person with SMI in case of FI [21, 22, 35, 36, 39]. Professionals consider confidentiality concerns a reason not to initiate FI as it could cause difficulties [22]. Especially in cases where persons with SMI are not consistent over time in their consent for sharing information [39], it is often unclear to professionals which information can be shared. Yet, even when persons with SMI consent to FI, professionals sometimes express concerns about *what kind* of information to share with families, especially when family relations are experienced as tense [39]. Therefore, even with consent, confidentiality is experienced as challenging in the context of FI.

### Barriers related to the family

*Persons with SMI* who address barriers related to the family encompass families not being available or unwilling to get involved [18], families who don’t understand the illness or find the illness difficult to accept [23, 24]. In several studies, persons with SMI report concerns regarding the negative consequences FI might have on their position and status within the family [21–23]. *Familie*s’ own concerns to FI include; not wanting to be involved, concerns about own privacy, wanting their discussions about care to be kept confidential from the persons with SMI [28], and experiencing discomfort when participating in group sessions with fellow families [26]. Also families report they do not want to intrude on the professional-patient relationship [21]. They mention low expectations regarding their own involvement as possibly helpful [26, 27], and report worries about whether they are the best person in the family to get involved [21]. Conflicts between work and other (family) commitments are reported as barriers for FI as well [21, 26]. *Professionals* report, repeatedly, that they experience difficulties in finding ‘suitable’ families for involvement, varying from persons with SMI who do not have ‘supportive’ family, to families interfering negatively with treatment [19, 34, 37, 41, 43, 45–47]. Families are considered a barrier if they are over-involved [43, 49] or lacking competence about mental illness or communication skills [26, 33, 41, 49]. Furthermore, professionals experience that families do not want to get involved [24, 33, 40–42, 47]. Next, concrete burdens of FI for the families or the wish to protect families against new or additional burdens are considered a barrier to FI [25, 40, 41]. Additional barriers are related to power-issues between families and professionals (regarding who knows best what is best for the person with SMI [22]) and/or families having a different cultural background [25, 49].

This second type of barriers; ‘family’ indicates similarities and differences in *how stakeholders experience each other as reliable.* Professionals report difficulties in finding ‘suitable’ families. Persons with SMI express worries that FI could jeopardize their autonomy within their families and can be afraid that FI may cause misunderstandings regarding their illness within their families [21–23]. Families themselves do not mention being unsuitable for FI explicitly. They experience often a lack of recognition and respect from professionals as main barrier for FI, which is described in the next paragraph.

### Barriers related to the professional

Only a few studies describe barriers related to professionals from the perspective of *persons with SMI*. One study reports that persons with SMI experience that professionals do not offer FI [18]. Another study, exploring perspectives of Latino-American persons with SMI report that professionals were hindering FI [25]. The majority of the studies that include *family* perspectives address barriers related to the professionals [22, 24, 25, 28–31]. Almost all report experiences of lack of recognition, respect and basic courtesy from professionals as large barriers to FI [24, 25, 28–31]. Families experience too much emphasis on confidentiality by professionals, which hinders FI when they are denied valuable information [24, 31]. Also the assumption of professionals that families should assume responsibilities for care for their relative without getting proper support is reported [31], as well as experiences of power struggles regarding who knows what is best for the person with SMI [22, 28]. *Professionals* report various barriers related to own concerns, e.g. insecurities and doubts regarding their own competence [35–37], as well as lack of experience [21, 24, 26, 36, 38]. One study describes poor sustainability of skills learned in training, and missing specialized background expertise [34]. For example, they find it difficult to deal with the emotions of families, and do not know how to deal with family issues. Also, professionals report they had low expectations of positive outcomes [33, 34], find it hard sometimes to motivate persons with SMI to participate [35], and found inter-professional struggles regarding which of the professionals should take the lead in FI [36].

In this overview of the ‘professional’ as type of barrier, it appears there are differences in expectations of *roles and responsibilities*. First, several studies report that professionals experience it as difficult themselves to determine what kind of role (responsibility) they have regarding families [21, 38] or which kind of professional (e.g. a psychiatrist, nurse or social worker) should take the lead [36]. At the same time, families expect more respect and support from professionals and lack recognition of their expertise and knowledge, which indicates they expect more involvement then professionals provide. Several studies report power struggles between professionals and families as barriers [21, 22, 28]. Second, professionals address themselves that they lack the skills, competence and experience to involve families [21, 24, 26, 35–38]. All these aspects illustrate that roles and responsibilities of the professional regarding FI are not straightforward and causes frictions. Besides, professionals frequently report that they do not experience support from their workplace to organize FI, which is described under the barrier related to the organization of care described below.

### Barriers related to the organization of care

*Persons with SMI* report a lack of flexibility of the organization of care to meet the schedules of families [18]. Barriers regarding the organization, described by *family*, include lack of available resources to realize FI, such as lack of time, locations to meet and lack of integration across different settings [24, 28]. *Professionals* address barriers related to the organization on a large scale. They report a lack of time to conduct FI [19, 21, 22, 33, 34, 36, 40, 44–47, 50], logistical barriers [24–26, 36, 38, 40, 41, 45, 46], and difficulties prioritizing FI in the organization [33, 34, 36, 43–45].

With the overview of this type of barrier, it becomes clear that the organization is not always experienced as supportive by all stakeholder perspectives in facilitating FI. Especially for professionals this causes concerns to what extent FI should be *prioritized* [30, 33, 34, 36, 43–45]. Professionals experience difficulties regarding the integration of FI with other responsibilities and tasks. Many reports of professionals refer to lack of time, having too many other demands, or no financial incentives as barriers to FI [19, 21, 22, 33, 34, 36, 40, 44–47, 50]. However, lack of time and resources to FI could also relate to lack of competence and skills among professionals (see above) or to the culture and paradigms (see below) among mental health care professionals.

### Barriers related to the culture-paradigm

*Persons with SMI* report stigma as barrier within their cultural context [24, 25]. Stigma and prejudices towards SMI hinder persons with SMI from involving others, such as family and friends. Language may hinder FI too, according to one study that included Latino-American persons with SMI [25]. *Families* also report stigma as barrier to FI [22, 24, 25, 32]. *Professionals* describe stigma as barrier as well, especially related to families with other cultural backgrounds [25, 33, 49]. Besides stigma, professionals report the medical paradigm as barrier [22, 43, 50]. Within the context and medically driven culture, there is a very strong focus on the individual patient, while families and social networks are generally perceived as secondary, and therefore often not included or prioritized in treatment [22]. Professionals also report management attitudes being unsupportive of FI [24, 40, 46]. Furthermore, professionals mention the influence of past experiences in one study [43], and that the technical language of professionals might not correspond to the language of families [22].

This overview of barriers illustrate that *stigma* is a barrier that is expressed as a concern by all the three stakeholders [24, 25, 32, 33, 49]. Stigma is a difficult concept to grasp as it can have different meanings for different stakeholders. In general, we assume that barriers of stigma addressed by the stakeholders express the fear of negative consequences of FI, such as exclusion from communities, and therefore hinders FI. This leads to the question how to reduce and cope with a context that is experienced as stigmatizing; how to empower families and persons with SMI to cope with stigma but also how to educate the public about SMI to reduce prejudices.

## Discussion

This review used a scoping review methodology in an innovative way by focusing on how various stakeholders conceptualize, perceive and understand barriers for family involvement (FI). We reported about the perspectives of three stakeholder groups: e.g. persons with SMI, their family and the professionals. In comparing the different stakeholder-perspectives on barriers for FI, we discovered similarities and differences regarding five main types of barriers. In analyzing and comparing the various stakeholder descriptions of barriers within each type of barriers, we discovered that the different stakeholder groups often have different perspectives and interests, and sometimes share the same presuppositions and experiences. For example, barriers related to persons with SMI, mainly centralized around the question how to handle confidentiality. While all stakeholders reported this as barrier to FI, they varied in how they experienced it as barrier and their perspectives on how the needs for confidentiality and privacy ought to be dealt with in practice. Second, the reported barriers related to families revealed a lack of trust between stakeholders. Perspectives correspond in experiencing distrust towards the other. Thus, a central question is how to develop mutual trust and understanding. In addition, the third type of barriers, related to professionals, illuminated the need for clear roles and responsibilities for all stakeholders. Especially professionals themselves were not sure what is expected of them regarding FI. Fourth, barriers related to the organization of care, illustrated that all stakeholder perspectives acknowledged practical difficulties in how to realize FI. They all missed clear guidance of the organization how much priority should be given to FI. However, our impression is that the families and also partly the person with SMI, give higher priority to FI than the professionals. Regarding the fifth type of barriers, the culture and paradigm of mental health care, all stakeholders acknowledged stigma as major hindrance for FI. The dominating paradigms – which often pay less attention to relational approaches and social networks- were also reported as an important barrier.

In the following, we will compare our findings with previous reviews on the topic of barriers regarding FI and discuss if and how our results add new insights to the debate why FI has not developed into a common practice in mental healthcare [2, 3]. A commonly recognized difficulty to implement evidence-based research into practice is that implementing new interventions often take much time. They require changes in working routines that are often confronted with resistance and challenges [51]. Translating research evidence into every day work is recognized as difficult [52]. Evidence of the beneficence of FI first needs to be believed by all participants involved. In the overview of barriers it is reported that stakeholders have low confidence in that FI can be helpful (persons with SMI [18]; family [26, 27]; professionals [33, 34]), which could explain the low uptake of FI. Several systematic reviews that have specifically looked into studies addressing barriers that hinder FI in practice, aligned with this conclusion. Mairs & Bradshaw [14] concluded that there was a lack of trust upon professionally developed and facilitated approaches. Eassom et al. [13] concluded that ‘top-down’ support and training in working with families is necessary but not sufficient, and advised that concerns such as privacy, power relations and fear of negative outcomes should be openly explored together in the triad including the patient, the family and the professionals. The findings of our review add to this debate in that it can give directions that are more specific in how to deal with barriers to FI. One main finding that emerged through this analytic approach is that differences in whether and how barriers are perceived can be in itself a barrier. If stakeholders do not talk about the barriers or about the differences in how they perceive barriers it is difficult to know about each other’s concerns. The thematic analysis of the barriers specifying how the various stakeholders experience the barriers deepens our understanding of how even the same barriers can have different interpretations.

Our findings illustrate that none of the stakeholders are neutral or passive participants. From all perspectives, barriers and conflict of interests are experienced. All stakeholders have own values and norms (either professional and/or personal) and interests that might cause specific barriers. To deal with these barriers in FI, this should be openly negotiated from the start of and during FI and discussed between the stakeholders as an inherent part of any FI intervention. Stakeholders should negotiate from the start and during FI how to deal with confidentiality and privacy. They could for example develop explicit agreements on which information could be shared and what should stay confidential and regularly mutually discuss if it works well for them. Second, how to create a context of mutual trust should be taken into account. Third, mutual responsibilities in FI should be openly discussed. The role of the professional should not to be a neutral facilitator, for example by giving systematic therapy to families, but should be recognized as one of the parties that has its own values and interests as well. Fourth, the importance and prioritization of FI has to be addressed and finally, awareness of the influence of stigma is relevant to empower families and persons with SMI to cope with those challenges.

## Strengths and limitations

In this scoping review, we specifically used the search methodology of Droste et al, to analyze different stakeholder perspectives on barriers. This innovative approach of reviewing abled us to compare different presuppositions and perceptions and distinguish domains wherein (moral) tensions may rise. It illustrates that different stakeholders experience differences in barriers and those differences can be a barrier in itself (addressing different moral domains). A major strength of this review is therefore that this methodology offered new insights in the (moral) complexities of FI.

One limitation is that the literature search was restricted to the English language, and therefore might have missed studies published in other languages. Second, the definitions of stakeholders were not formulated in a strict sense. We followed the definitions of the studies in how they used this ordering. What counts as family, for example, is open for debate. It could be argued that if family is not involved in the lives of the persons with SMI, or acknowledged as ‘important other’ for the person with SMI, it should not be counted as family. These nuances could not be extracted from the studies that used the stakeholder perspectives in their data. However, we are confident that the stakeholder perspectives represent barriers that spring from their roles, values and interests. Third, studies that include persons with SMI and families require voluntary cooperation of these stakeholders. Therefore, findings are possibly biased, as not all persons with SMI and families would want to participate in research. Many of the included studies describe the study limitation that their findings might have limited generalizability. Fourth, the studies included in the review covered a period of more than two decades (1993-2015). It is possible that (moral) attitudes and perspectives have changed during that period and different barriers might be experienced between then and now. We did not specifically focus on the historical context and moral backgrounds of the studies in our analysis but it would be an interesting topic to follow up and to further deepen the findings.Fifth, we did not specifically address the quality of the studies and the methodologies used therein, as we were searching for any possible barriers that described by the different stakeholder groups, rather than to assess the quality or validity or the results (e.g. to make it possible to compare outcomes of intervention studies)

## Conclusions

Our review is novel compared to existing literature on this topic in that it reveals differences in stakeholder perspectives regarding which barriers are experienced and how they are experienced. It also clearly reveals that different stakeholders have different conceptions, presuppositions, interests, motives and expectations regarding FI, and regarding the others involved, due to their specific context, experiences and backgrounds. These differences can be a barrier in itself if the stakeholders do not talk about the barriers and acknowledge the possible differences in how they perceive barriers. Awareness of how barriers are experienced by stakeholders is important to understand the actual dynamics that hinder the uptake and quality of FI and to foster critical reflection regarding the barriers. For example, further investigation regarding the barrier of the lack of time experienced by professionals may illuminate a relationship with lack of resources, lack of competencies or lack of priority within the dominating paradigm or in the organization, which again may influence the perception of roles and responsibilities.

To deal with barriers to FI a dialogical approach is required, in any FI intervention that stimulates explicit identification and discussion of the various barriers, perceptions and interests. It should include all stakeholders in concrete situations to foster mutual understanding, better collaboration and balancing of possible conflicts of interest.

## Recommendations

We recommend a continuous dialogue between the stakeholders from the start – in any FI intervention - to discuss how they perceive FI and what they experience as barriers. In practice, explicit identification of all barriers, their meanings, related presuppositions, underlying values and ideals should be scrutinized. If not, stakeholders may remain ‘worlds apart’.

Explicit identification requires an open attitude towards the other. Specific methods that take into account various conceptions and norms regarding FI, such as moral case deliberation and systematic ethics reflection can be beneficial to find ways to bridge and overcome the differences. Further research in how to use these methods most optimal in the context of FI is recommended. Research should specifically address how these methods can address the questions illuminated in the analyses of this review, e.g. 1) how to handle confidentiality and privacy, 2) the need for mutual reliability, 3) clarification of roles and responsibilities, 4) which priority to give FI, and 5) the influence of stigma. To stimulate the use of FI in practice, further research could contribute with developing blueprints and strategies to facilitate the dialogue in triad.

## Additional file

Additional file 1: Detailed description of the search strategy. (DOCX 15 kb)

## Abbreviations

FI: Family involvement
SMI: Severe mental illness
