# Supplementary material for: Worlds apart? A scoping review addressing different stakeholder perspectives on barriers to family involvement in the care for persons with severe mental illness
Source: BMC Health Serv Res. 2017 May 15;17:349. doi: 10.1186/s12913-017-2213-4 (PMC5433083; doi:10.1186/s12913-017-2213-4)
Supplement: Additional file 1: — Detailed description of the search strategy. (DOCX 15 kb) [file 12913_2017_2213_MOESM1_ESM.docx]

Additional file 1: Detailed description of the search strategy

Medline via Ovid

1. exp affective disorder, psychotic/ or exp bipolar disorder/ or exp psychotic disorders/ or exp schizophrenia/
2. (psychos* or psychotic or schizo* or (bipolar adj disorder)).tw.
3. ((severe or serious) adj (mental* or psychological or psychiatric) adj (disorder* or ill* or problem*)).tw.
4. 1 or 2 or 3
5. (challenge* or barrier* or difficult* or dilemma* or obstacle*.tw.
6. exp confidentiality/ or exp duty to warn/
7. ((services adj families) or (working adj1 families) or (engaging adj1 families) or (alliance adj1 families adj1 professional*)).tw.
8. exp Professional-Family Relations/
9. ((social adj support) or (informal adj care) or triad* adj care* or (family adj care) or famil*adj2 decision-making)).tw.
10. ((family or families or relatives or carer* or friend* or (social adj network) or (personal adj network)) adj2 (therapy or psychotherapy or psychoeducation or counseling or work or treatment or intervention or management or approach or involve* or incl* or participate*)).tw.
11. 7 or 8 or 9 or 10
12. 5 or 6
13. 4 and 11 and 12
14. Limit 13 to (abstracts and English language)

PsycINFO via Ovid

1. exp schizoaffective disorder/ or exp schizoid personality disorder/ or exp schizophrenia/ or exp ‘schizophrenia (disorganized type)’ / or schizophreniform disorder/
2. exp psychosis/
3. (psychos* or psychotic or schizo* or (bipolar adj disorder)).tw.
4. ((severe or serious) adj (mental* or psychological or psychiatric) adj (disorder* or ill* or problem*)).tw.
5. 1 or 2or 3 or 4
6. (challenge* or barrier* or difficult* or dilemma* or obstacle*).tw.
7. ((social adj support) or (informal adj care) or triad*adj care* or (family adj care) or famil* adj2 decision-making)).tw.
8. ((family or families or relatives or carer* or friend* or (social adj network) or (personal adj network)) adj2 (therapy or psychotherapy or psychoeducation or counseling or work or treatment or intervention or management or approach or involve* or incl* or participate*)).tw.
9. 7 or 8
10. 5 and 6 and 9
11. limit to (all journals and abstracts)

Cinahl via ESBSCO

S1. (MH “Psychotic Disorders+”) OR (MH “Affective Disorders, Psychotic+”) OR (MH “Schizophrenia+”)

S2. TI (psychos*s or psychotic or schizo* or (bipolar W0 disorder))

S3. TI ((severe or serious) W0 (mental* or psychological or psychiatric) W0 (disorder* or ill* or problem*))

S4. AB ((severe or serious) W0 8mental* or psychological or psychiatric W0 (disorder* or ill* or problem*))

S5. AB (psychos*s or psychotic or schizo* or bipolar W0 disorder))

S6 S1 OR S2OR S3 OR S4 OR S5

S7 TI (challenge* or barrier* or difficult* or dilemma* or obstacle*)

S8 AB (challenge* or barrier* or difficult* or dilemma* or obstacle*)

S9 S7 OR S8

S10 TI ((services W0 families) or (family W0 service*) or (working W0 with W0 families) or (engaging N0 families) or (alliance N0 families N0 professional*)).tw.

S11 TI ((services W0 families) or (family W0 service*) or (working W0 with W0 families) or (engaging N0 families) or (alliance N0 families N0 professional*))

S12 AB ((services W0 families) or family W0 service*) or (working N0 families) or (engaging N0 families) or (alliance N0 families N0 professional*))

S13 TI ((social W0 support) or (informal W0 care) or triad* W0 care* or (family W0 care) or (famil* N1 decision-making))

S14 AB ((social W0 support) or (informal W0 care) or triad* W0 care* or (family W0 care) or ((famil* N1 decision-making))

S15 TI ((family or families or relatives or carer* or friend* or (social W0 network) or personal W0 network)) N1 (therapy or psychotherapy or psychoeducation or counseling or work or treatment or intervention or management or approach or involve* or inclu* or participate*))

S16 AB ((family or families or relatives or carer* or friend* or (social W0 network) or personal W0 network)) N1 (therapy or psychotherapy or psychoeducation or counseling or work or treatment or intervention or management or approach or involve* or inclu* or participate*))

S17 S10 OR S11 OR S12 OR S13 OR S14 OR S15 OR S16

S18 S6 AND S9 AND S17

S19 S6 AND S9 AND S17 limiters – Abstract Available; English Language

Social Science Citations Index via Web of Knowledge

**TOPIC:** ((("severe mental") OR ("serious mental") NEAR (disorder* OR illness OR problem*)) OR (("severe psychiatric") OR ("serious psychiatric") NEAR (disorder* OR illness OR problem*)) OR schizophrenia OR ("psychotic disorder*") OR ("bipolar disorder*")) *AND* **TOPIC:** ((services NEAR families) OR ("working with families") OR ("engaging with families") OR ("social support") OR ("informal care*") OR ("triad* care*") OR ("family care*") OR ("family decision-making") OR ("family involvement") OR ("family intervention*") OR ("family participat*") OR (family psychoeducation) OR (family counselling)) *AND* **TOPIC:** (challenge* OR barrier* OR difficult* OR dilemma* OR obstacle*)
Refined by: **DOCUMENT TYPES:** (ARTICLE OR REVIEW)
